# Endothelial progenitor cells for fabrication of engineered vascular units and angiogenesis induction

**DOI:** 10.1111/cpr.13716

**Published:** 2024-07-25

**Authors:** Somayyeh Rashidi, Ghasem Bagherpour, Zahra Abbasi‐Malati, Nafiseh Didar Khosrowshahi, Sara Aghakhani Chegeni, Golbarg Roozbahani, Hamid Lotfimehr, Emel Sokullu, Reza Rahbarghazi

**Affiliations:** ^1^ Department of Medical Biotechnology, Faculty of Medicine Zanjan University of Medical Sciences Zanjan Iran; ^2^ Zanjan Pharmaceutical Biotechnology Research Center Zanjan University of Medical Sciences Zanjan Iran; ^3^ Student Research Center Tabriz University of Medical Sciences Tabriz Iran; ^4^ Stem Cell Research Center Tabriz University of Medical Sciences Tabriz Iran; ^5^ Chemical Engineering Faculty Sahand University of Technology Tabriz Iran; ^6^ Department of Clinical Biochemistry and Laboratory Medicine Tabriz University of Medical Sciences Tabriz Iran; ^7^ Department of Plant, Cell and Molecular Biology, Faculty of Natural Sciences University of Tabriz Tabriz Iran; ^8^ Research Center for Translational Medicine (KUTTAM) Koç University Istanbul Turkey; ^9^ Biophysics Department Koç University School of Medicine Istanbul Turkey; ^10^ Department of Applied Cell Sciences, Faculty of Advanced Medical Sciences Tabriz University of Medical Sciences Tabriz Iran

## Abstract

The promotion of vascularization and angiogenesis in the grafts is a crucial phenomenon in the healing process and tissue engineering. It has been shown that stem cells, especially endothelial progenitor cells (EPCs), can stimulate blood vessel formation inside the engineered hydrogels after being transplanted into the target sites. The incorporation of EPCs into the hydrogel can last the retention time, long‐term survival, on‐target delivery effects, migration and differentiation into mature endothelial cells. Despite these advantages, further modifications are mandatory to increase the dynamic growth and angiogenesis potential of EPCs in in vitro and in vivo conditions. Chemical modifications of distinct composites with distinct physical properties can yield better regenerative potential and angiogenesis during several pathologies. Here, we aimed to collect recent findings related to the application of EPCs in engineered vascular grafts and/or hydrogels for improving vascularization in the grafts. Data from the present article can help us in the application of EPCs as valid cell sources in the tissue engineering of several ischemic tissues.

## Graphical abstract

The current review article highlights the angiogenesis properties of endothelial progenitor cells as valid cell sources for the induction of angiogenesis using various tissue engineering modalities. The application of different substrates with several synthesis protocols was also discussed in detail.

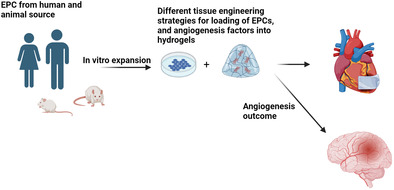

## 1 INTRODUCTION

**FIGURE 1:**
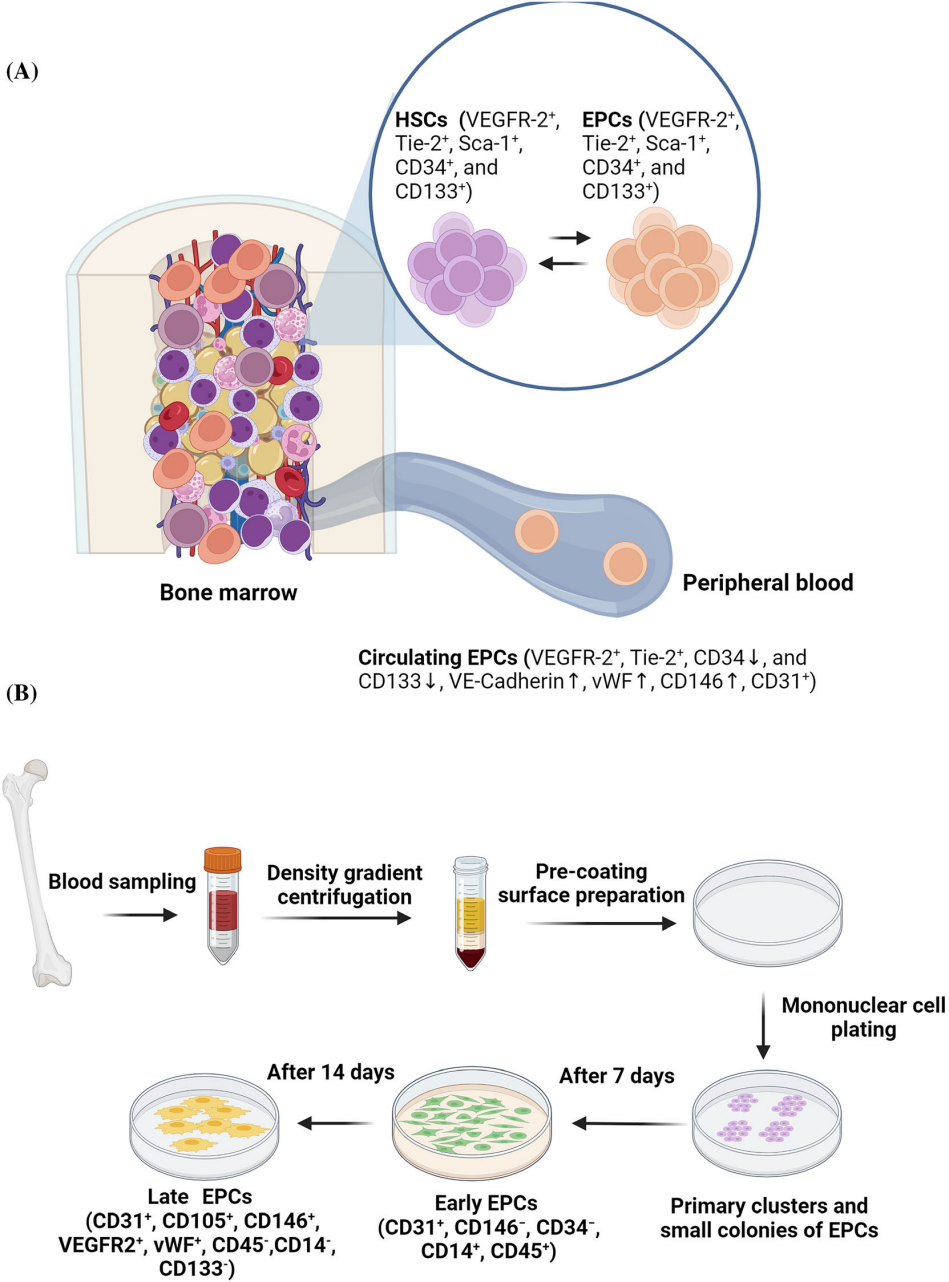
Immunophenotypic similarities between bone marrow endothelial progenitor cells (EPCs) and haematopoietic stem cells (HSCs) (A). The EPC surface markers are changed after entering the systemic blood circulation. Procedure of EPC culture, morphological and immunophenotypic changes in in vitro culture systems (B).

The phenomenon of angiogenesis and vascularization needs the participation of several cell types, mainly endothelial lineages, such as mature endothelial cells (ECs) and endothelial progenitor cells (EPCs), which can support the development and progression of vascular beds across almost all tissues under physiological and pathological conditions.^1^ Recent data have indicated that EPCs can differentiate into mature ECs to repopulate the ECs at the site of injury. Besides, EPCs are potent cell sources to release several angiogenesis factors, leading to the promotion of blood support into the hypoxic/ischemic areas via a paracrine manner.^2^ Like other stem types, EPCs possess self‐renewal properties and differentiation capacity for the healing of injured tissues and ischemic organs via vasculogenesis. It seems that the differentiation potential of these cells is not limited to the endothelial lineage. Some data showed the direct orientation of EPCs towards cardiac pericytes and mural cells.^3^ EPCs are bone marrow‐residing stem cells and originate from hemangioblasts.^4^, ^5^ In response to angiogenesis factors gradients, EPCs enter the blood circulation and promote the formation of de novo vessels via oriented maturation into mature ECs.^4^, ^6^, ^7^, ^8^ Since hemangioblasts are a common source of EPCs and haematopoietic stem cells (HSCs), molecular immunophenotyping techniques have revealed the existence of several common surface markers such as Flk‐1 (KDR or VEGFR‐2), Tie‐2, Sca‐1, CD34 and CD133 in EPCs and HSCs, making it difficult to obtain the pure source for different regenerative purposes (Figure 1A).^9^

To date, several studies have been conducted to isolate, characterise and immunophenotype the expanded EPCs in different animal species and human counterparts (Table 1). Bone marrow is the main site of EPCs, but these cells can also be found in the blood as circulating ECs or in a small number in other tissues.^51^ Thus, bone marrow, peripheral blood and umbilical cord blood are the main biological samples for the isolation of EPCs.^52^ Other proposed tissues and niches for the isolation of EPCs include the vascular bed niche, placenta, spleen, adipose, cardiac and neural tissues.^53^ However, the exact origin of non‐bone marrow EPCs is unclear and needs further investigation. In adults, bone marrow is the main tissue source for obtaining EPCs, but umbilical cord blood usually provides higher EPC numbers with prominent stemness features (telomerase activity↑), and proliferation capacity for biobanking and clinical applications.^54^ In terms of angiogenesis properties, umbilical cord blood EPCs exhibited long‐term and robust tubulogenesis capacity compared to EPCs isolated from other sources. Meanwhile, the isolation of umbilical cord blood EPCs is relatively non‐invasive, with less possibility of infection transmission and graft‐versus‐host disease.^54^ It has been proposed that embryonic stem cells (ESCs) are an alternative source for harvesting and expansion of EPCs in vitro.^55^ Compared to umbilical cord blood EPCs, ESC‐derived EPCs exhibited a higher proliferation rate and efficiently promoted ischemic wound healing in mice by the release of fibroblast growth factor‐2 (FGF‐2), vascular endothelial growth factor (VEGF), angiopoietin‐1 (Ang‐1) and induction of angiogenesis.^55^ Like ESCs, induced pluripotent stem cells have been used for the production of EPCs and further maturation into functional venous and arterial ECs.^56^ In this regard, Rosa et al. produced EPCs (CD31^+^/KDR^+^/VE‐cadherin^+/−^/EphB2^−^/COUP‐TF^−^) from human K2‐iPSCs. The procedure was continued with the differentiation of EPCs towards venous and arterial ECs. Data showed appropriate function of both EC types in response to vasoactive compounds (thrombin and prostaglandin E2) similar to somatic ECs.^56^ Despite the existence of different EPC origins, there is no consensus in terms of standard protocols and tissue sources for regenerative purposes.

**TABLE 1:** Some studies used different markers for the isolation of endothelial progenitor cells (EPCs) in different species.

EPCs immunophenotyping and characterisation	Species	Sample	Isolation method	References
CD34^+^/vWF^+^/PECAM+/thrombospondin^+^	Human	BM	Ficoll density gradient centrifugation	10
CD34^+^/KDR^+^/CD31^+^/eNOS^+^/E‐selectin^+^	Human	PB	Magnetic bead selection	5
CD34^+^/Flk‐1^+^/vWF^+^	Human	BM, CB, FL and PB	Ficoll density gradient centrifugation	11
CD34^+^/KDR^+^/CD133+	Human	CB, PB and FL	Magnetic bead selection	12
KDR^+^/VE‐cadherin^+^/CD31^+^	Human	PB	Histopaque density gradient centrifugation	13
KDR^+^/VE‐cadherin^+^/Flt‐1^+^/eNOS^+^/vWF^+^	Human	PB	Histopaque density gradient centrifugation	14
CD133^+^/CD34^+^/KDR^+^/vWF^¯^/VE‐cadherin^¯^/CD31^¯^	Human	BM	Ficoll density gradient centrifugation	15
CD31^+^/Tie 2^+^/VEGFR2^+^	Human	PB	Ficoll density gradient centrifugation	16
CD31^+^/VEGFR‐2^+^/Tie‐2^+^/VE‐cadherin^+^/eNOS^+^/CD14^¯^	Human	PB	Histopaque density gradient centrifugation	17
VEGFR2^+^/Tie‐2^+^/VEGFR‐1^¯^	Human	PB	Ficoll density gradient centrifugation	18
KDR^+^/VE‐cadherin^+^/CD31^+^/eNOS^+^/Flt‐1^+^/vWF^+^	Human	PB	Histopaque density gradient centrifugation	19
CD31^+^/VE‐cadherin^+^/eNOS^+^/VEGFR2^+^	Mouse	BM	Ficoll density gradient centrifugation	20
CD34^+^/CD45^¯^/VEGFR2^+^/CD133^¯^	Human	CB and BM	Lymphoprep density gradient centrifugation	21
CD34^+^/CD45^¯^	Human	CB or PB	Fluorescence‐activated cell sorting	22
vWF^+^/CD31^+^/CD144^+^	Porcine	PB	Histopaque density gradient centrifugation	23
PECAM‐1^+^/EDN‐1^+^/Flk‐1^+^/vWF^+^/ITGAD^+^/CCR^−^1^+^/IP30^+^/MMP‐2^+^	Rat	PB	Histopaque density gradient centrifugation	24
CD31^+^/CD34^+^/CD144^+^/vWF^+^	Human	UCB	Density gradient centrifugation	25
CD34^+^/VEGFR2^+^/CD133^+^/CD31^+^/vWF^+^	Human	UCB	Percoll density gradient centrifugation	26
CD31^+^/CD105^+^/CD146^+^/CD45^¯^/CD14^¯^/CD34^+^/CD117^+^/CD133^¯^	Human	PB	Density gradient fractionation	27
CD133^low^/FLK1^+^/CD31^+^	Rat	BM	Ficoll density gradient centrifugation	28
CD31^+^/CD34^+^/CD144^+^/CD146^+^/Flt‐1^+^/Flk‐1^+^/Flt‐4^+^/Nrp2^+^	Human	CB	Ficoll density gradient centrifugation	29
CD34^+^/CD133^+^/VEGFR‐2^+^	Chicken	BM	Percoll density gradient centrifugation	30
eNOS^+^/Flk1^+^/VE‐cadherin^+^	Mouse	PB	Histopaque density gradient centrifugation	31
CD133^+^/VEGFR‐2^+^/CD31^+^/CD34^¯^	Chicken	PB	Lymphoprep density gradient centrifugation	32
CD34^+^/CD133^+^/CD31^+^	Rhesus monkey	BM	Lymphoprep density gradient centrifugation	33
CD31^+^/CD146^+^/VEGFR2^+^	Rat	BM	Histopaque density gradient centrifugation	34
CD31^+^/CD34^+^/VEGFR2^+^/αSMA^−^	Human	UCB	Density gradient centrifugation	35
CD34^+^/CD31^+^/CD73^+^/CD105^+^/VEGFR2^+^/CD45 ¯/CD90 ¯	Human	UCB	Direct collagenase digestion	36
VEGFR2^+^/eNOS^+^/vWF^+^/CD31^+^/VE‐cadherin^+^	Rat	BM	Histopaque density gradient centrifugation	37
CD45^−^/CD31^+^/CD34^+^/CD105^+^/CD146^+^/CD157^±^/CD200^±^	Human	AEPCs	Magnetic bead selection	38
Flk‐1^high^/CD31^+^/VEGF^+^/Flt‐1^+^/ANGPT1^+^/ANGPT2^+^	Human	BM	Grinding the bone and centrifugation	39
CD31^+^/CD14 ¯	Porcine	PB	Density gradient solution	40
DiI‐AC‐LDL uptake, FITC‐UEA‐I uptake, CD31^+^/CD34^+^/vWF^+^/VEGFR‐2^+^	Porcine	Femoral artery puncture	Histopaque density gradient centrifugation	41
DiI‐AC‐LDL uptake, CD31^+^, VEGFR‐2^+^	Human	PB	Isolation of CD34^+^ cells using magnetic beads	42
FITC‐UEA‐I binding and DiI‐AC‐LDL uptake	Human	CB	Ficoll density gradient centrifugation	43
CD31^+^/CD34^+^/CD133^+^/VEGFR‐2^+^, FITC‐UEA‐I binding and DiI‐AC‐LDL uptake, Matrigel tubulogenesis	Canine	BM	Direct culture of bone marrow cells	44
Weibel–Palade bodies imaged by TEM	Rabbit	PB and BM	Biocoll density gradient	45
CD133^+^/CD34^+^/VEGFR‐2^+^/vWF^+^	Mouse	CB	Magnetic bead selection (CD133)	46
CD34^+^/VEGFR2^+^/CD133^+^/CD31^+^	Mouse	BM	Density gradient centrifugation	47
CD34^+^/VEGFR‐2^+^/CD133^+^, FITC‐UEA‐I binding and DiI‐AC‐LDL uptake, karyotyping	Bovine (foetus)	BM	Density gradient centrifugation	48
CD31^+^/VEGFR‐2^+^, Geltrex matrix tubulogenesis	Human	AEPCs	Enzymatic digestion and centrifugation of stromal vascular fractions in lipoaspirate samples	49
CD14^−^/CD31^+^/CD34/CD45^−^/CD105^+^/CD117/CD141^+^/CD144^+^/CD146^+^/VEGFR‐2^+^/vWF^+^	Human	BM and CB	CD34^+^ cells were injected into NOD/SCID mice to obtain high‐proliferative potential ECFCs	50

Abbreviations: αSMA, alpha smooth muscle actin; AEPCs, adipose‐derived EPCs; BM, bone marrow; CB, cord blood; Dil‐AC‐LDL, DiI‐labelled acetylated low‐density lipoprotein; ECFCs, endothelial colony‐forming cells; eNOS, endothelial nitric oxide; FL, foetal liver; FITC‐UEA‐I, fluorescein isothiocyanate‐labelled Ulex Europaeus Agglutinin I; NOD/SCID, nonobese diabetic/severe combined immunodeficiency; PB, peripheral blood; TEM, transmission electron microscope; UCB, umbilical cord blood, vWF, von willebrand factor.

Upon entry into the systemic circulation, EPCs are characterised based on the existence of the common hemangioblast makers CD34, CD133 and VEGFR2.^8^, ^12^, ^57^ In contrast to the bone marrow niche, the levels of stemness markers such as CD133 are reduced in the blood, especially with the maturation of ECs.^12^, ^57^, ^58^ The same phenomenon can occur during the in vitro culture of EPCs, leading to the loss of stemness features and endothelial phenotype acquisition.^4^, ^59^ Based on in vitro data, both changes in the morphology and molecular profile of EPCs are evident over time.^22^, ^60^, ^61^, ^62^ Along with these changes, the distribution of mature EC markers such as VE‐cadherin, vWF and CD146 increases.^63^ During the first 4–10 days of culture in a laboratory setting, early EPCs (CD31^+^, CD146^−^, CD34^−^, CD14^+^ and CD45^+^) are spindle shape and transform into late EPCs (CD31^+^, CD105^+^, CD146^+^, VEGFR2^+^, vWF^+^, CD45^−^, CD14^−^ and CD133^−^) with cobblestone morphologies after 14 days (Figure 1B).^64^, ^65^, ^66^, ^67^

**FIGURE 2:**
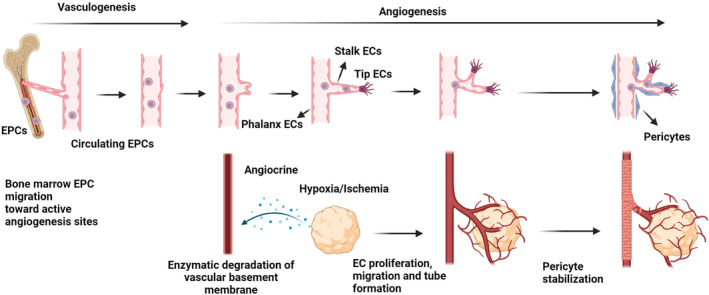
Angiogenesis and vasculogenesis steps for the generation of de novo blood vessels. In response to ischemia and hypoxia, cells release angiocrine with different pro‐angiogenesis cytokine panels. In the angiogenesis phenomenon, the attachment of these factors to endothelial cells (ECs) in the proximity can result in the activation of vascularization. Following EC activation, these cells secrete proteolytic enzymes to degrade the supporting basal membrane and undergo proliferation. The leading ECs at the tip of new blood vessels (Tip ECs) migrate towards the hypoxic zone in cytokine gradient density. Stalk ECs can maintain EC‐to‐EC tight junctions to assure the stability of new sprouts, resulting in the formation of the vascular lumen. With the elongation of vascular units towards the target sites, stalk ECs become quiescent, which are named phalanx ECs. Besides angiogenesis, in vasculogenesis, bone marrow endothelial progenitor cells (EPCs) are recruited after the production of several angiogenesis factors. These cells enter the systemic blood circulation and become circulating EPCs with a gradual loss of stemness markers. After homing to the target sites, EPCs can mature into ECs and trans‐differentiate into other cell lineages. The final step of angiogenesis is the stabilisation and wrapping of newly generated vascular units by pericytes (also known as arteriogenesis).

In in vivo conditions, the phenomenon of new blood formation is done via two distinct mechanisms, as follows: angiogenesis and vasculogenesis (Figure 2).^68^, ^69^ The term angiogenesis, or neo‐angiogenesis, encompasses all cellular and molecular mechanisms for the production of new blood vessels from pre‐existing vascular networks.^70^ While vasculogenesis is the main new blood vessel formation during the embryonic period.^70^ Of course, it should not be forgotten that vasculogenesis is observed during adulthood with the participation of EPCs to restore blood supply to the injured area or healing tissues.^71^ To promote angiogenesis and generate new vessels, ECs produce sprouts in response to pro‐angiogenesis factors such as VEGF, FGF, etc. ECs lose their connection with the neighbour ECs and produce extracellular matrix (ECM)‐degrading enzymes to remodel the angiogenesis niche. The ECs are close to the hypoxic microenvironment and start to migrate in a gradient of cytokine factor and the acquisition of specific EC phenotype, namely tip cells, resulting in guiding the blood vessels towards affected sites (Figure 2).^72^

Other vascular ECs differentiate into stalk and phalanx cells based on their location and interaction with different cytokines.^73^ Previous data have revealed that tip cells are highly motivated cells with prominent migration capacity and help the vascular trunk generate buds. Unlike tip ECs, stalk ECs exhibit high‐rate proliferation capacity and are involved in the formation of vascular lumens.^74^, ^75^ Unlike these EC phenotypes, phalanx ECs are relatively silent without proliferation capacity and participate in vascular structure stability.^76^, ^77^, ^78^

Studies have indicated that EPCs can be isolated from bone marrow (haematopoietic or myeloid EPCs), blood (circulating EPCs), the umbilical cord and other tissues like fat, such as nervous and dental tissue, etc.^79^ Myeloid and circulating EPCs can foster the vascularization in a paracrine manner releasing several angiogenesis factors, or direct maturation into functional ECs.^80^, ^81^

## 2 THE EMERGENCE OF ENGINEERED VASCULAR UNITS

In recent decades, attempts have focused on the fabrication of engineered vascular units using several substrates loaded with different vascular cells.^82^ The existence of ethical issues, immune system reactivity in response to allogenic and xenogeneic vascular grafts, and a lack of suitable autologous sources limit the application of natural blood vessels in patients with cardiovascular diseases (CVDs).^83^, ^84^ In general, autologous vascular grafts are isolated from arteries related to mammary tissue, radial and thoracic arteries and saphenous and brachial veins. In most circumstances, the existence of certain concomitant pathologies, a lack of appropriate blood vessel length, etc. can prohibit the use of autologous vascular tissue for transplantation purposes.^84^ Studies have also reported the possibility of thrombosis, atherosclerotic plaques, infections (cytomegalovirus) and even intimal hyperplasia (arterial thickening↑) at the site of transplantation.^85^ Of course, it should not be forgotten that the type and intensity of angiopathies after vascular conduit transplantation can differ from endogenous vasculitis under pathological conditions. For example, it was suggested that accumulated lipids are less and exhibit a diffuse pattern compared to atherosclerotic conditions. In contrast to massive calcification in atherosclerosis, seldom calcified foci can be detected.^86^ Of note, the phenomenon of transplant rejection is mainly mediated by the activity of circulating monocytes, in which the number of monocyte–macrophage lineages can reach nearly 60%–70% of total immune cells at the site of transplantation. The activity of macrophages at the site of transplantation leads to the promotion of an acute inflammatory response.^87^ A few days after transplantation, blood CD14^+^ monocytes can penetrate transplantation sites and commit directly into both macrophages (M1 [inflammatory], M2 [anti‐inflammatory] types) or dendritic cells via the modulation of specific factors MafB, PU.1 and IRF4, AHR, respectively.^87^ The activity of macrophages and dendritic cells leads to the increase of local inflammatory cytokines (IL‐12↑) and recruitment of T lymphocytes (CD4^+^ and CD8^+^ cells), resulting in allograft rejection.^87^ M1 macrophages predispose the acute vascular graft rejection via the production of IL‐1, −6, −12, 23, CCL2, CXCL9, 10 and 11, while M2 macrophages increase the possibility of fibrosis via the release of IL‐10, TGFβ1 and CCL18.^87^

During the last century, human medicine witnessed a splendid improvement in the design and production of artificial vascular conduits. For example, artificial products composed of synthetic polyesters such as polytetrafluoroethylene (PTFE, Teflon®) and polyethylene terephthalate (PET; Dacron®) have been used for vascular surgeries.^88^ Despite their unique physicochemical properties, these polymers also contribute to uncontrolled thrombosis, hyperplasia of intimal layer cells, the formation of atherosclerotic plaques and proliferation of fibroblasts and tunica media alpha‐smooth muscle actin (α‐SMA^+^) cells.^85^ These effects would be related to the inability of artificial vascular units to promote the proliferation and formation of the endothelial layer at the luminal surface.^89^ Using novel and sophisticated technologies, such as the application of porous scaffolds with specific surface modification, it is possible to activate the ECs at the edge of natural blood vessels to migrate towards transplanted grafts and furnish the surface. Of note, researchers have not been able to successfully modify human and other mammalian natural vascular units because it increases the possibility of gradual breakdown and the formation of aneurysms.^90^ Besides, the application of Dacron‐ and Teflon‐based vascular conduits has been limited in coronary artery bypass surgery due to a lack of suitable physicochemical properties when used as small‐sized diameter vascular conduits.^91^ These features prohibit the bulk application of artificial vessels in paediatric patients.^91^

### 2.1 Manufacturing methods

**FIGURE 3:**
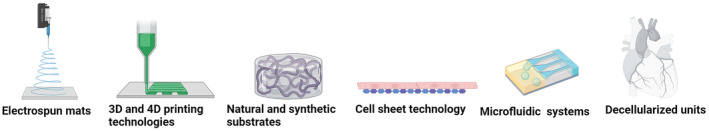
Different fabrication modalities are applied to promote the angiogenesis properties of engineered units.

Commensurate with these descriptions, the design and development of biocompatible vascular conduits with features near native blood vessels have been at the centre of attention in tissue engineering and regenerative medicine.^91^ To be specific, engineered vascular conduits are composed of supporting scaffolds with plated vascular cells that coincide with the stimulation of certain signalling pathways for better regenerative outcomes.^91^ First, collagen (Col) was used for the preparation of vascular conduits, and transplanted grafts resisted only for short periods.^92^ Thus, researchers have used other modalities such as hybrid substrates, decellularized units, cell sheet engineering, 3D printing technologies, electrospun mats and multi‐part microfluidic channel systems to improve the physicochemical properties of vascular graft (Figure 3).^82^

The electrospinning approach enables us to produce porous and fibrous scaffold mats, while the fibre diameters, porosity intensity and 3D structure of substrates can be precisely modulated. By combining diverse natural and synthetic components using different formulas, vascular conduits with specific physicochemical and biological properties can be achieved.^93^, ^94^, ^95^, ^96^ In vascular tissue engineering based on decellularisation, all vascular cells are eliminated while scaffold‐like ECM remains relatively intact with the ability to promote cell dynamic growth and proliferation via providing suitable topography and biochemical cues.^93^, ^95^ Using 3D and 4D bioprinting techniques, it is possible to make regular cell layers within the applied biometric in specific dimensions. It is postulated that this approach is beneficial for the production of personalised vascular conduits using patient‐derived cells with specific biomaterials.^93^, ^95^ In an interesting experiment, cytocompatible engineered vascular conduits were fabricated using the microfluidic bioprinting technique.^97^ To this end, the dual network was developed with a bio‐ink prepolymer composed of alginate, gelatin (Gel), Gel methacryloyl (GelMA) and bacterial transglutaminase, along with an ionic cross‐linker CaCl_2_. To mimic venous‐like vascular structure, umbilical vein ECs and smooth muscle cells (SMCs) were plated in the luminal and outer surfaces. In the arterial structure, the outer umbilical SMC layer encapsulated the inner hydrogel‐containing umbilical artery ECs. The arterial conduits exhibited suitable vascular constriction and dilation in response to 10 μM phenylephrine and/or acetylcholine, respectively.^97^ The incubation of engineered vascular units with SARS‐CoV‐2 particles led to viral infection due to the prominent and proper expression of angiotensin‐converting enzyme‐2 in ECs.^97^ Data confirmed that the designed engineered vascular units were appropriate preclinical in vitro platforms for the evaluation of several pathological conditions. The 3D bioprinting technique enables us to fabricate complex personalized tissue microstructures with interconnected vascular networks, resulting in timely anastomosis with the host vessels of grafts and an accelerated healing process.^98^ Liu and co‐workers developed an interconnected 3D capillary engineered microstructure using EC‐laden inks (GelMA and fibrinogen) with enzymatic reaction (thrombin) and photo‐crosslinking in the presence of lithium phenyl‐2,4,6‐trimethylbenzoylphosphinate and ultraviolet irradiation (UV).^98^ The synthesis protocol included sol–gel transition with decreasing ambient temperature, application of UV irradiation to cross‐link GelMA and polymerisation of fibrinogen by the addition of thrombin.^98^ Under a dynamic culture system with a continuous flow rate (2–20 μL/min), human umbilical vein ECs (HUVECs) along with mesenchymal stem cells (MSCs) generated CD31^+^ capillary‐like networks. Using this technique, vascularized hepatic tissue organoid was fabricated using HUVECs, HepG2 and foreskin fibroblasts at a ratio of 30%, 60% and 10%, respectively. Data showed the upregulation of hepatic and vascular tissue‐specific factors such as albumin and CD31.^98^ Taken together, this approach is useful for the fabrication of centimetre‐scale complex interconnected vascularized units for different regenerative purposes.^98^ In another study, Wang et al. assessed the angiogenesis properties of HUVECs in composites consisting of GelMA microgels embedded inside 3% GelMA +0.25% fibrin with phosphosilicate calcium bioactive glass. Data indicated that the designed platform possesses suitable mechanical stability, supporting HUVEC migration to different parts of the hydrogel. Confocal imaging revealed the localisation of HUVECs at the luminal surface of vascular beds and a suitable EC‐to‐EC juxtacrine interaction. The transplantation of printed engineered vascular structure within the poly(dimethylsiloxane) to arteries in rats led to blood perfusion after several days as indicated by successful anastomosis with host vascular beds.^99^ Therefore, the combination of different disciplines such as tissue engineering, material science, immunology and regenerative medicine approaches can help the production of functional vascular units with high‐rate self‐interactive and ‐regenerative properties in patients.^93^, ^94^, ^95^ In the context of biomaterial, attention should be taken to the application of biocompatible substrates with suitable biomechanical properties in engineered vascular units is mandatory.^82^ Besides, using appropriate synthesis techniques, vascular grafts with unique topographies and shapes (tubular structure), distinct pore sizes and fibre alignment patterns can be fabricated.^100^, ^101^ As mentioned above, the possibility of thrombosis formation limits the application of vascular units in clinical settings.^102^ In this regard, some studies have been conducted based on the application of anti‐clotting agents such as heparin to fabricate blood‐compatible surfaces.^102^

### 2.2 Materials for engineered vascular units

During recent years, engineered vascular conduits were fabricated using natural and synthetic polymeric scaffolds composed of PTFE, PET, polyurethanes (PU), polycaprolactone, poly(lactic‐co‐glycolic acid), poly(lactic‐co‐lactic acid), polylactic acid (PLA), polyglycolic acid, poly(lactic‐co‐caprolactone), polyethylene glycol (PEG), polyethylene oxide and poly(lactide‐co‐ε‐caprolactone).^100^, ^103^ The use of natural substrates such as Col, elastin, fibrin, Gel, hyaluronic acid, silk and chitosan is common in the structure of engineered vascular units for improving the biological effects.^95^, ^104^, ^105^ Because of functional groups and motifs, natural ECM components and substrates can increase adhesion and stimulate the seeded cells.^105^, ^106^ Among natural substrates, Col has been extensively used in engineered vascular units due to its abundance in the ECM and the feasibility of extraction.^107^, ^108^ Noteworthy, synthetic polymers have fewer biological properties compared to natural substrates compared to engineered vascular units with specific synthesis formulas. Despite the superiority of engineered vascular conduits over synthetic grafts, the application of engineered vascular conduits faces some limitations, as follows: design of large‐scale vascular units (*x* >1 cm^3^) is problematic and needs specific technology. Alignment and spatial arrangement of cells within the composite are another critical issue.^109^ The fabrication of long‐lasting small‐diameter vascular units with minimum thrombogenicity is difficult.^100^ The same story is evident for synthetic grafts, in which these products exhibit short‐lasting periods and the possibility of thrombus formation, hyperplasia and stenosis is high in small‐diameter‐sized units. The orientation and arrangement of vascular cell lineages, such as EPCs are also laborious and difficult.^94^ Thus, the selection of an appropriate substrate for the fabrication of engineered vascular conduits with comparable physicochemical properties and biological effects is crucial.^110^ The recent advances have led to the production of suitable engineered vascular units capable of clinical settings.^100^ Also, the engineered vascular grafts have not matched the performance of autologous vessels, and there are challenges in developing clinically appropriate vascular grafts.^94^

The attachment of EPCs to the underlying substrate is a critical step in the development of vascularized engineered units. It has been thought that the components, design and manufacturing of hydrogels can affect the physiological behaviour of EPCs.^111^ In an experiment, electrospun Gel (10%) with an average diameter of 333 ± 130 nm was used for human EPC culture and expansion.^112^ Data showed that cultured EPCs can be aligned along with nanofiber direction and express relevant biomarkers such as CD133 and CD31 several days after plating.^112^ These data indicated that regulation of cell‐to‐substrate interaction, and spatial alignment can pre‐determine the angiogenesis properties of EPCs within the final composites. Like the size of nanofibers, the interconnectivity of the pores can also affect the cell response. The culture of EPCs on 3D printed PLA substrates with different pore sizes (1.27 ± 0.06 and 0.7 ± 0.02 mm) led to a reduction of vWF and delayed cell proliferation compared to MSCs.^113^ Thus, the type of cells, physicochemical properties of substrates and 3D microstructure can affect the dynamic growth of transplant cells and regenerative potential. Of note, decellularized scaffolds with intact 3D structures are also applicable for the induction of vasculogenesis at the transplantation site.^111^ The co‐culture of rat EPCs with decellularized kidneys led to round‐shape morphology, phenotype acquisition (CD133^+^↑) and an increase of proliferation (BrdU^+^↑) after 3 days.^111^ Orthotopic transplantation of acellular kidneys in rats led to the development of vascular anastomosis, with the localisation and repopulation of CD31^+^ and α‐SMA^+^ cells within the renal sinus, leading to the generation of renal vessels.^111^ Some strategies are based on the co‐loading of growth factors along with EPCs to obtain better angiogenesis outcomes. In an interesting study, porcine small intestinal submucosa was functionalized with a cyclic αvβ3 integrin ligand, namely LXW7, Col‐binding peptide (SILY), along with dermatan sulphate to increase the angiogenesis properties of rat EPCs. The transplantation of fabricated substrate to diabetic rats with a skin flap model led to the generation of neoepidermis, Col production, survival of endothelial lineage and angiogenesis potential (RECA‐1^+^ and α‐SMA^+^ cells).^114^ These data show that the application of functionalized scaffolds with the potential to increase EPC, or vascular cell attachment, can accelerate the process of angiogenesis. Taken together, the regulation of endothelial lineage function can be achieved via the regulation of transplant cell attachment to the supporting substrates using ECM components or the addition of adhesive moieties via chemical and physical approaches.

## 3 APPLICATION OF HYDROGEL‐COATED EPCs IN ANGIOGENESIS

### 3.1 Application of hydrogel‐loaded cytokines in EPC angiogenesis

**FIGURE 4:**
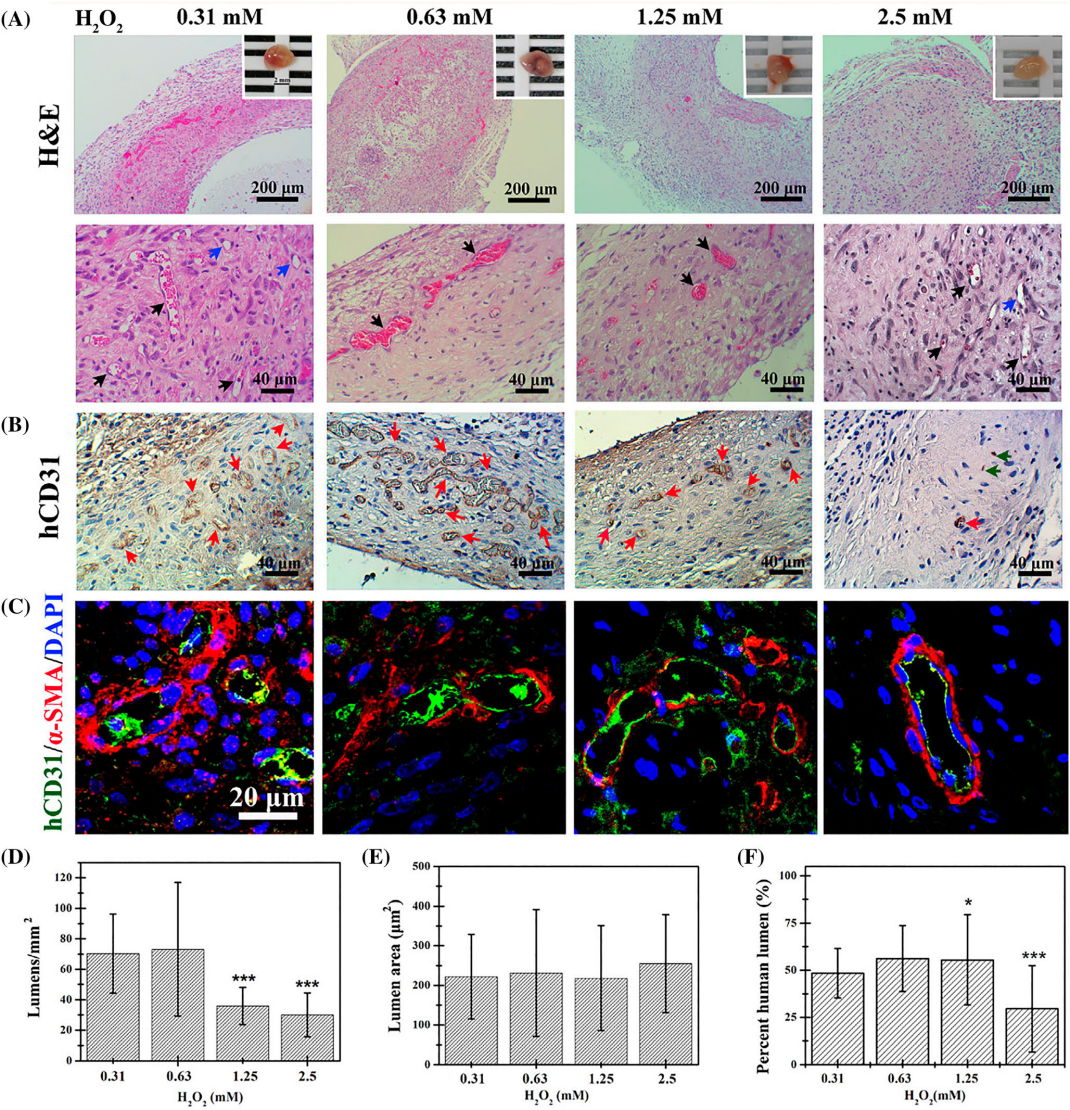
Monitoring angiogenesis properties of human mesenchymal stem cells (MSCs) (1.2 × 10^6^) and endothelial colony‐forming cells (0.8 × 10^6^) embedded inside phenolated porcine Gel hydrogel (200 μL) in the presence of 0.078 U/mL horseradish peroxidase and different concentrations of H_2_O_2_ (0.31, 0.63, 1.25 or 2.5 mM) 7 days after transplantation in nude mice (A–F). The manipulated hydrogel with target cells was injected subcutaneously into nude mice. Hematoxylin‐Eosin staining of phenolated cell‐loaded Gel hydrogel with various degrees of crosslinking (A: low magnification: top panels, and high magnification: bottom panels). Macroscopic view: scale bar: 2 mm. In the bottom panels, black and blue arrows indicate perfused and non‐perfused vascular units, respectively. Immunohistochemistry analysis for the detection of CD31^+^ cells (B). Red arrows are identical to perfused vascular units furnished with CD31^+^ cells. Immunofluorescence images of green‐coloured Alexa Fluor® 488 CD31^+^ ECs and red‐coloured Alexa Fluor® 594 α‐SMA^+^ perivascular cells (C). Anti‐CD31 antibody can only react with transplanted human cells. The background staining was done using 4′,6‐diamidino‐2‐phenylindole (DAPI). Measuring microvascular density by the calculation of erythrocyte‐containing vascular units (D–F). Microvascular density was calculated in histological sections (D), lumen size of vascular units (E) and CD31^+^ blood vessel percent (F). The experiments were done in quadruplicate to sextuplicate. Data are compared to the hydrogel cross‐linked with 0.31 mM H_2_O_2_. *p < 0.05; and ***p < 0.001.^136^ Copyright 2015. Acta Biomaterialia.

According to several studies, EPCs have been used for different regenerative purposes due to their inherent properties to promote vascularization into the ischemic sites (Table 2).^115^ Hydrogels with the ability to mimic the 3D ECM structure have been used for the fabrication of several engineered vascular units.^148^, ^149^ Hydrogels with polymeric network structures can absorb considerable amounts of water, which facilitates the reciprocal interchange of nutrients between the grafts and surrounding host tissues.^150^ Flexibility, cytocompatibility, injectability, etc. are other important features that make hydrogel suitable for different tissue engineering purposes.^128^ Using several technical approaches, hydrogel‐based scaffolds can be fabricated with certain physicochemical properties and different sizes.^151^ According to several previously published articles, homogenous ECs, or heterogenous cell populations can be incorporated into scaffold structure before the gelation process or added to a pre‐made porous structure. Of note, the first approach can yield evenly distributed cells and relatively similar angiogenesis activity within the hydrogel structure compared to the direct plating of cells on the surface of pre‐fabricated structures.^127^, ^152^ Due to the cytocompatibility of hydrogels and mimicking of a 3D ECM‐like structure, it is possible to use EPCs and mature ECs as particular cell sources for the generation of vascular tissue in in vivo and in vitro conditions.^153^ Previous studies have used chemical or physical methods to cross‐link the polymeric network composed of natural and synthetic substrates. Natural hydrogels such as natural polysaccharides (chitosan, sodium alginate, hyaluronic acid, agarose, etc.) and proteins (tropocollagen, Col, Gel, silk fibroin, etc.) have been used for the fabrication of engineered vascular units.^151^, ^154^, ^155^ Chuang et al. reported that phenolated Gel hydrogel from porcine sources is a suitable platform for the induction of angiogenesis.^136^ The developed hydrogel exhibited higher stiffness and storage modulus using different concentrations of H_2_O_2_ (0.31, 0.63, 1.25 or 2.5 mM) and 0.078 U/mL horseradish peroxidase. Co‐injection of human cord blood endothelial colony‐forming cells (ECFCs) and adipose tissue MSCs loaded with phenolated Gel into subcutaneous spaces in nude mice led to appropriate vasculogenesis. Immunofluorescence data revealed the incorporation of Alexa 488 CD31^+^ ECs and Alexa 594 α‐SMA^+^ cells into the structure of vascular units (Figure 4).^136^ In an experiment, photo‐cross‐linkable EPC‐ and acidic FGF (aFGF)‐loaded methacrylate‐Gel hydrogel stimulated wound closure, re‐epithelialization (pan‐cytokeratin↑) and induction of HIF‐1α in rats with diabetic ulcers, indicating angiogenesis‐related healing process during the diabetic conditions.^136^ In vitro data also confirmed the stimulatory effects of EPC‐/aFGF‐loaded methacrylate‐Gel hydrogel on HUVEC survival rate and migration.^136^ In vascular tissue engineering, the phenomenon of endothelialization is touted as a critical step for achieving appropriate vasculogenesis.^131^, ^156^ Strategies based on EPC recruitment and capture can help in in‐time homing of these cells and endothelialization. For example, the development of biomaterials with enhanced ligand‐receptor affinity is useful for EPC‐related endothelialization. For instance, Camci‐Unal et al. indicated the selectivity of anti‐CD34 coated methacrylated hyaluronic acid (1% w/v)/methacrylate‐Gel (2% w/v) substrate in adhering human cord blood EPCs compared to other cell lineages such as macrophages.^131^ The attachment of cells onto the surface of fabricated scaffolds seems like a fundamental step in acquiring a functional phenotype in which the loss of cell attachment to the supporting surface can lead to growth arrest and apoptosis.^128^ It was suggested that porous methacrylate‐Gel microspheres with porogen particles enhanced mouse EPC attachment, typical morphological properties, survival rate (calcein AM^+^ cells) and vascularization (vWF^+^ and VE‐cadherin^+^ cells).^128^ In some studies, binding domains and motifs are synthetically added to the structure of hydrogel to promote cell attachment and morphological adaptation.^128^ It seems that the incorporation of ECM epitopes such as Arg‐Gly‐Asp‐Ser (RGDS), arginylglycylaspartic acid (RGD), appropriate ligands for cell integrins and SIKVAV (laminin α1‐derived peptide) has benefits to accelerate EC proliferation and alignment (tubulogenesis↑).^157^

**TABLE 2:** Different studies related to the application of several substrates and endothelial progenitor cells (EPCs) for angiogenesis potential.

Hydrogel	Type of cells	Modifier and growth factor	Animal	Method of fabrication	Type of injury	Outcome	References
Chitosan	Human CB EPCs	‐	Rat and sheep	Lyophilization	CVD	Biocompatibility↑ and haemocompatibility↑	115
Star PEG‐heparin	Human PB EPCs	Variant of SDF‐1α		Polymerisation	AMI	Long‐term delivery of growth factor and apoptosis↓	116
Hyaluronic acid	Mouse BM EPCs	Polymeric RGD	Mouse	Crosslinking	KI	Long‐term EPCs release	117
HA/Alginate/heparin	Human CB EPCs and HUVECs	Anti‐CD34 Ab		Lyophilization	CVD	EPC adhesion and spreading↑	118
Alginate	Human CB EPCs	GGGGRGDSP oligopeptides using charcoal	CAM	Lyophilization	CVD	Controllable degradation and swelling	119
Acryl‐PEG	Rat BM EPCs and NSCs	RGDS GGGGGPQGIWGQGG‐Lys‐[alloc]‐GK peptide	Rat	Lyophilization	SCI	Cell spreading↑ and interneuron connection after grafting↑	120
GelMA	Rat BM EPCs	Sr^2+^ ions for incorporation of RGD	Rat	Freeze‐drying	Wound healing	Swelling↑, mechanical characteristics↑, self‐healing↑, biodegradation↑, biocompatibility↑, EPC migration↑, proliferation↑ and adhesion↑	121
GelMA/alginate	HUVECs		Mice	Electrospraying	Hindlimb ischemia	Stable mechanical properties, angiogenic activity↑	122
Dextran	Mouse BM EPCs		Nude mouse	Crosslinking	CVD	ECM‐like 3D culture	123
Col/chitosan	HUVECs		Mouse	Syringe mixing system	CVD	Resistant to enzymatic degradation↑, denaturation↓, endothelial cell differentiation↑ and angiogenesis↑	124
PEG	Rat BM EPCs	RGD, VEGF and bFGF	CAM	Lyophilization	Ischemic orthopaedic diseases	Mechanical properties↑, suitable for a sustained release purpose	125
HPLG/fibrinogen	HUVEC and GPC		CAM	Ionic gelation	Bone tissues repairing	HUVEC viability↑, sprouting↑ and mechanical properties↑	126
GelMA	Mouse EPCs			Photo‐crosslinking	CVD	Gel‐MCG density improves homogenous vascularization	127
GelMA	Mouse EPCs			Photo‐crosslinking	CVD	EPC adhesion↑, proliferation↑, endothelial differentiation↑ and vascularization↑	128
PEGDA/alginate	Rat BM EPCs		Mouse	Photo‐crosslinking	Diabetic wound healing Skin repairing	Injectability↑, Self‐healing↑, viscoelasticity↑, mechanical and antibacterial properties↑, cytocompatibility↑, angiogenesis↑	129
PEG	HUVECs/10T1/2 MSCs	GGGPQG↓IWGQGK RGDS VEGF	Mice	Photo‐crosslinking	Cornea injury	Proteolytically‐degradability↑, cell adhesion↑, angiogenesis↑	130
GelMA/HA‐MA	Human CB EPCs	CD34 Ab		Photo‐crosslinking	CVD	Cell adhesion↑, spreading and morphological elongation↑, biocompatibility↑	131
HA	Rat BM EPCs	ESA	Rat	Lyophilization	MI	Degradability↑, long‐term factor release, angiogenesis↑, left ventricular function↑	132
GelMA	Human PB ECFCs/MSCs		Mouse	Photo‐crosslinking	CVD	Formation of functional vascular networks tunable mechanical and chemical properties	133
Fibrin	Rat BM EPCs		Rat		MI	Enhance cell delivery cell retention and vasculogenesis	134
HA	Rat BM EPCs		Rat	Lyophilization	Ischemic heart disease	Enhanced cell retention and vasculogenesis	135
Gel‐Ph	Human CB MSCs		Mouse	Enzyme‐based crosslinking	CVD	Cytocompatibility↑, tunable mechanical properties↑, proteolytic degradability↑	136
Col‐Ph	Human PB EPCs and MSCs		Mouse	Enzyme‐based crosslinking	CVD	Controllable physicochemical properties, vascularization↑	137
PEGDA	Human PB EPCs HUVECs	Scl2‐2 protein		Photo‐crosslinking	CAD	Resistance to platelet adhesion, activation and migration	138
Alginate‐PLO‐Gel	Human CB EPCs	Magnetic nanoparticles	Mouse	Layer‐by layer technology	Wound healing	EPC migration↑, vascularization↑ and dermal wound repair↑	139
Acryl‐PEG‐SVA	Human CB EPCs/SMCs	RGDS GGGPQG↓IWGQK		Photo‐crosslinking	CVD	3D microvessel formation↑	140
Pullulan/dextran	Human CB EPCs	Fucoidan polysaccharide VEGF	Mouse	Chemically crosslinking	Ischemic injury	Vascular density↑ and angiogenesis↑	141
Alginate/Gel	Human CB EPCs/MSCs	SDF‐1α		Crosslinking		Tubulogenesis↑	142
Chitosan	Human CB EPCs and BM MSCs		Rat	Freeze‐drying	CVD	Elasticity↑, cell adhesion↑ and Vascularization↑	143
Chitosan/graphene oxide	Human CB EPCs			Freeze‐drying	CVD	Cell proliferation↑ and angiogenesis potential↑	144
PEGDA	Human CB EPCs	RGDS RGES RGDSHHHHHHG YIGSRG and REDV		Photo‐crosslinking	CVD	Biocompatibility↑ and ECFC rolling↑	145
PEG/fibrinogen	PB EPCs		Horse	Photo‐crosslinking	Limb wound healing	Survival rate and proliferation↑	146
PEG/fibrinogen	Huma PB EPCs	rhBMP2	Rat	Photo‐crosslinking	Tibia defects	Sustained delivery of rhBMP2	147

Abbreviations: Acryl‐PEG‐SVA, acrylate‐PEG‐succinimidyl valerate; AMI, acute myocardial infarction; bFGF, basic fibroblast growth factor; BM, bone marrow; CAD, coronary artery disease; CAM, chick chorioallantoic membrane; CB, cord blood; Col, collagen; CVD, cardiovascular disease; ESA, engineered stromal cell‐derived factor analogue; Gel, gelatin; Gelatin‐Ph, gelatin‐phenolic hydroxyl; GelMA, Gel methacryloyl; GPCs, gingiva‐derived progenitor cells; HA, hyaluronic acid; hBMSCs, human bone marrow mesenchymal stem cells; HPLG, human platelet lysate gel; HUVECs, human umbilical vein endothelial cells; KI, kidney injury; MC, micro‐cavitary gel; MI, myocardial infarction; NSCs, neural stem cells; PB, peripheral blood; PEG, poly(ethylene glycol); PEGDA, poly(ethylene glycol)diacrylate; PLO, poly‐L‐ornithine; RGD, rginylglycylaspartic acid; RGDS, Arg‐Gly‐Asp‐Ser; rhBMP2, recombinant human bone morphogenic protein‐2; SCI, spinal cord injury; SMCs, smooth muscle cells; VEGF, vascular endothelial growth factor.

Of course, it should not be forgotten that the encapsulation of vascular cells inside the microspheres and hydrogels can limit their migration and function.^158^ Thus, fabrication and designing of degradable scaffolds are mandatory in terms of appropriate angiogenesis and vascularization into ischemic areas. Iatrogenic addition of degrading enzymes (i.e., collagenases) is also applicable to control embedded cell migration capacity after being transplanted into the targeted sites.^159^ In an experiment conducted by Moon et al., they produced PEG‐based hydrogel conjugated with metalloproteinase 2 and 9 (MMP‐2 and ‐9)‐sensitive peptide (GGGPQG↓IWGQGK) and adhesion ligand motif [Arg‐Gly‐Asp‐Ser (RGDS)] and VEGF factor.^130^ They found that the designed hydrogel can foster in vitro tubulogenesis properties of multipotential 10T1/2 stem cells and HUVECs. Besides, direct differentiation of 10T1/2 cells towards the mural lineage with the production of type IV Col and laminin was also reported, resulting in the stability of vascular units.^130^ The release of VEGF from MMP‐sensitive PEG hydrogel stimulated the formation of blood vessels in mice cornea using micropocket angiogenesis assay.^130^ It should not be forgotten that embedding stem cells inside the injectable hydrogels not only provides a delivery platform for reaching the cells into the injured sites but also can protect the cells against direct noxious stimuli and insulting conditions.^159^ Ratliff et al. used embryonic EPC‐loaded hyaluronic acid hydrogel for the stimulation of angiogenesis under different pathological conditions in mice with adriamycin‐induced nephropathy, renal ischemia and hindlimb ischemia.^160^ They declared that the addition of collagenase and hyaluronidase grants the timely release of embedded EPCs into the injured sites, leading to tightly controlled angiogenesis outcomes. Of note, the lack of degrading enzymes lasts the EPC retention inside the hydrogel for several weeks.^160^ Previous data have confirmed that the incorporation of certain cytokines such as VEGF, FGF, along SDF‐1α inside the hydrogel can concomitantly stimulate angiogenesis and migration properties of encapsulated cells.^132^

Fabrication of hydrogels containing protease‐sensitive peptides is another strategic approach for timely induction of angiogenesis.^130^ For instance, MacArthur et al. used HiLyte Fluor‐labelled SDF‐1α analogue namely engineered stromal cell‐derived factor analogue (ESA) peptide inside the injectable hydrogel composed of hydroxyethyl methacrylate‐modified hyaluronic acid for the stimulation of EPC angiogenesis properties.^132^ Data indicated the sustained released of loaded ESA from hydroxyethyl methacrylate‐modified hyaluronic acid substrate near 4 weeks in in vitro conditions. These features increased the chemotaxis of rat EPCs in the Transwell insert® system, indicating the functionality of loaded ESA inside the injectable hydrogel.^132^ The injection of fluorescent‐tagged ESA in rats with myocardial infarction led to sustained release of loaded peptide for 24 days, leading to improvement in cardiac tissue output and fraction ejection. Histological examination indicated the reduction of fibrotic changes and enhanced vascularization indicated by the recruitment of CXCR4^+^ and vWF^+^ cells into the site of ischemia.^132^ In an experiment, Saberianpour et al. studied the angiogenesis potential of human CD133^+^ EPCs co‐incorporated with MSCs inside the alginate (1% w/v)‐Gel (2% w/v) microspheres in the presence of 10 ng/mL SDF‐1α.^161^ They found that microsphere supplementation with SDF‐1α delayed the maturation of EPCs to mature ECs indicated by the reduction of VE‐cadherin and tubulogenesis capacity coincided with the induction of MMP‐9 especially when co‐cultured with MSCs.^161^ These data highlight the fact that caution should be taken when specific cytokines are added to final composites in terms of angiogenesis. Without considering the appropriate concentrations of chemoattractant cytokines, the migration of embedded EPCs may be stimulated, while on the other hand maturation and phenotype acquisition of these cells can be also postponed. Following the ischemic conditions, the release of SDF‐1α from injured sites and further attachment to the surface CXCR4 receptor stimulate EPC migration in a cytokine gradient manner. Thus, sustained release of SDF‐1α promotes angiogenesis and vascularization into injured sites.^162^ Commensurate with these descriptions, SDF‐1α is one of the most important cytokines that can be used in final hydrogels to increase angiogenesis potential. Of note, SDF‐1α can directly attach to glycosaminoglycans via ionic interactions. Thus, hydrogel‐containing glycosaminoglycans such as hyaluronic acid can preserve SDF‐1α for long periods without changes in its structure and function.^162^ On the other hand, hyaluronic acid can make physical contact with the EPC CD44 receptor and improve the migration properties.^162^

The application of bioengineering solutions with different tissue engineering modalities can help us develop a highly sophisticated complex polymeric network relatively similar to the native vascular tissue niche.^163^ The addition of several signalling biomolecules improves the physiological activity of loaded cells or helps the host cells recruit into the target sites. Fabrication of hydrogels armed with spatial cues can dictate specific angiogenesis behaviour for EPCs and vascular cells after being transplanted into the target tissues. Using advanced techniques, such as 3D and 4D bioprinting, with certain levels of growth factors and EPC numbers within the distinct geometries can lead to more prominent angiogenesis potential.^164^ Bioprinting and electrospinning with patterned bioactive molecules can improve the direction of endothelial lineage in in vitro and in vivo conditions.^164^

### 3.2 Application of hydrogel‐loaded growth factors in EPC angiogenesis

Besides the incorporation of EPCs into the final composites, some experiments have focused on the addition of pro‐angiogenesis factors alone or in combination with endothelial lineage before grafting into the target sites to increase the angiogenesis potential.^161^, ^165^ Based on the great body of previous scientific data, different molecular interactions are commonly used to stabilise the growth factors for sustained release and prolonged activities inside the body.^166^, ^167^ Unfortunately, most applied growth factors possess short half‐lives and limited stability due to high levels of proteolytic enzymes and thermal degradation in in vivo conditions, which influence the bioactivities after being transplanted into the injured sites.^161^, ^168^ In a better word, the burst release of loaded factors just a few hours and/or days after transplantation led to massive distribution, rapid deactivation of these factors and a lack of regenerative response in long‐term periods. Thus, it seems that the application of hydrogels can prolong the factor release in a biphasic and triphasic manner.^168^ Of note, the inherent physicochemical properties of loaded growth factors can pre‐determine their stabilities from a few minutes to hours and days after being incorporated into the final composites. For example, basic fibroblast growth factor (bFGF), a potent angiogenesis stimulator, possesses an in vivo half‐life of about 3 min and these values are about 16 min and 1 h for bone morphogenetic protein‐2, and NGF, respectively.^169^ Growth factors with larger molecular sizes exhibit difficulties in delivery and access to the injured sites.^169^ It is noteworthy to mention that the load of higher angiogenesis factors in transplanting hydrogels can increase the risk of tumour formation, abnormal vascular tissue development, prominent edema and systemic hypotension.^169^ It is believed that the incorporation of growth factors inside the hydrogels can protect them from the direct activity of tissue enzymes, and regulate their release. These features can reduce the total required dose and the possibility of unwanted side effects in the target organs.^169^

**FIGURE 5:**
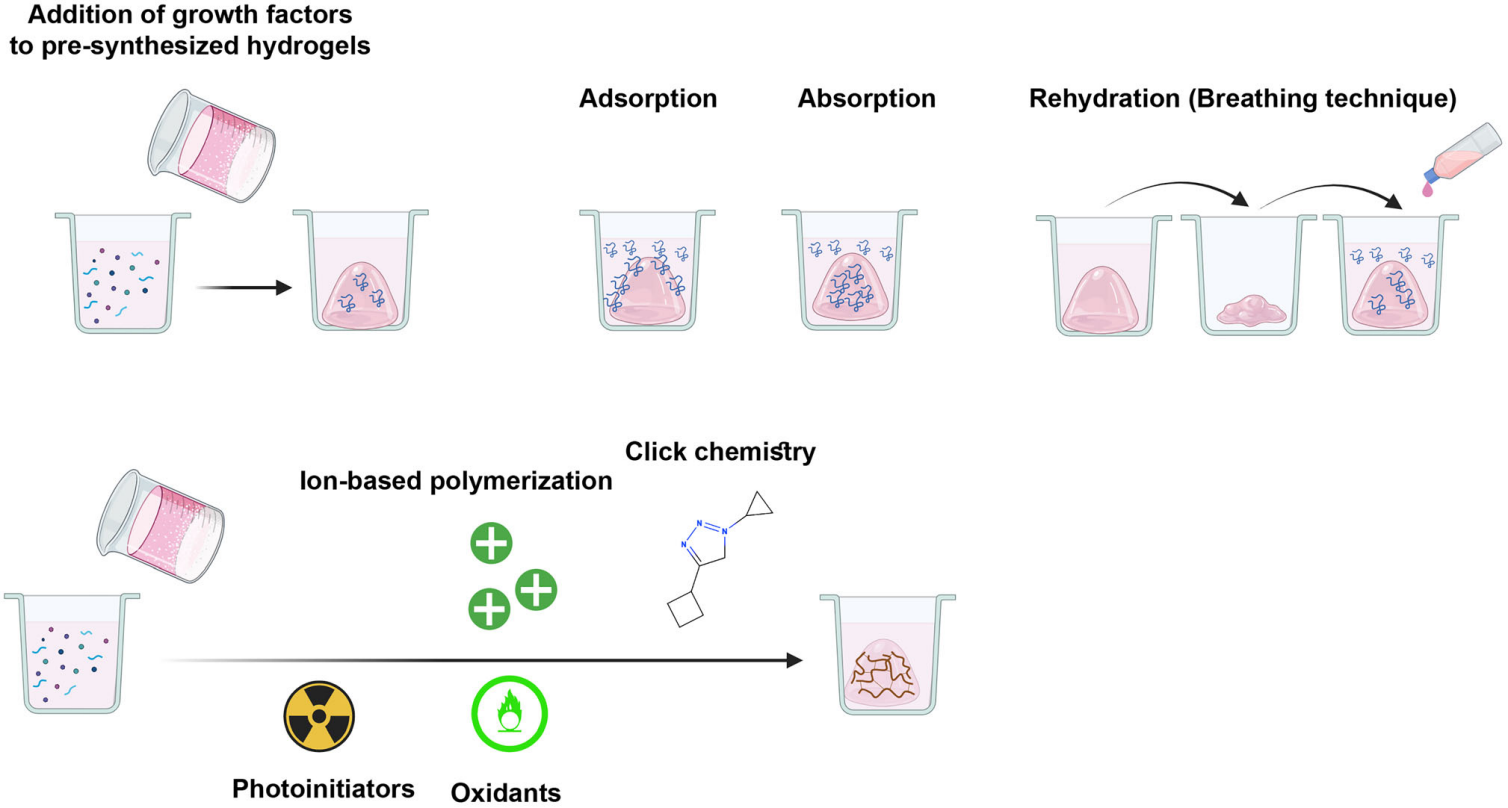
Different physical and chemical interactions are commonly used for the loading of angiogenesis growth factors into or surface of final graft composites. Growth factors and cytokines can be added to the pre‐polymer solution and dissolved before the initiation of the polymerisation step, which is also known as the pre‐loading method. The post‐loading strategies are based on the addition of certain growth factors and cytokines to pre‐synthesized hydrogels, which include absorption, adsorption and rehydration (breathing) methods. Adsorption is defined as the phenomenon in which soluble factors, cytokines and growth factors are transferred from the liquid phase onto the surface of solid hydrogel due to the existence of physical forces or chemical reactions. This reaction is commonly reversible (desorption) and can lead to equilibrium between attached factors on the hydrogel surface and soluble content in the liquid phase. Temperature is a fundamental element that affects the adsorption rate. Compared to adsorption, in absorption, the targeted factors, or therapeutics can enter or distribute to deep layers or hydrogel. This feature correlates with an initial concentration of growth factors, the existence of functional groups within the polymeric network, porosity and crosslinking network's density within the hydrogel. Rehydration loading allows the entry of growth factors into the deep layer of hydrogel. Using covalent reactions (click chemistry, free radicals and photoinitiators), growth factors are cross‐linked with the hydrogel network, leading to the long‐term release of these factors. The ionic crosslinking of growth factors is reversible compared to the chemically covalent reactions.

There are several synthesis strategies for the addition of certain growth factors to the polymeric network of hydrogels. Specific growth factors can be loaded into hydrogel before (pre‐loading), and after (post‐loading) polymerisation.^170^ In the pre‐loading approach like encapsulation, growth factors dissolve in the aqueous phase before the initiation of polymerisation, while in the latter modality, such as absorption, adsorption and rehydration (also known as the breathing technique), the target growth factors are added to pre‐synthesised hydrogels.^170^ It should not be forgotten that each method has its advantages and disadvantages. Direct exposure to chemical compounds can increase the possibility of degradation and oxidation of growth factors because of free radical‐mediated crosslinking techniques and photoinitiators (i.e., UV, etc.). Under such conditions, the application of non‐radical mediated crosslinking approaches has superiority (Figure 5).^171^ Having porosity and swelling features, it allows growth factors to easily penetrate the polymeric networks in rehydration and absorption methods. Compared to absorption, lyophilized hydrogels are applied to the load of targeted growth factors in the rehydration method.^172^ In absorption, the targeted growth factors penetrate the deep layer of the final composites, while these factors are attached to the surface of hydrogel using adsorption techniques.^173^ In general, chemical and physical approaches can be used for the load of growth factors to the final composites.^174^ In physical entanglement, incorporated growth factors are sequestrated inside the polymeric networks or surface without the formation of any bonds with hydrogels. This approach can lead to the burst release of loaded factors during early times after being transplanted into the target sites. In contrast, the generation of chemical bonds between certain growth factors and supporting hydrogels (covalent and non‐covalent binding) can affect the releasing time more than that of features described in physical methods.^175^ In chemical modifications, covalent interactions are mechanically stable and generated using different methods such as carbodiimide chemistry, glutaraldehyde crosslinking, and transglutaminase. Compared to covalent chemical interactions, non‐covalent chemical modifications are induced via electrostatic interactions between entrapped growth factors, and proteins with polymeric hydrogels.^176^

**FIGURE 6:**
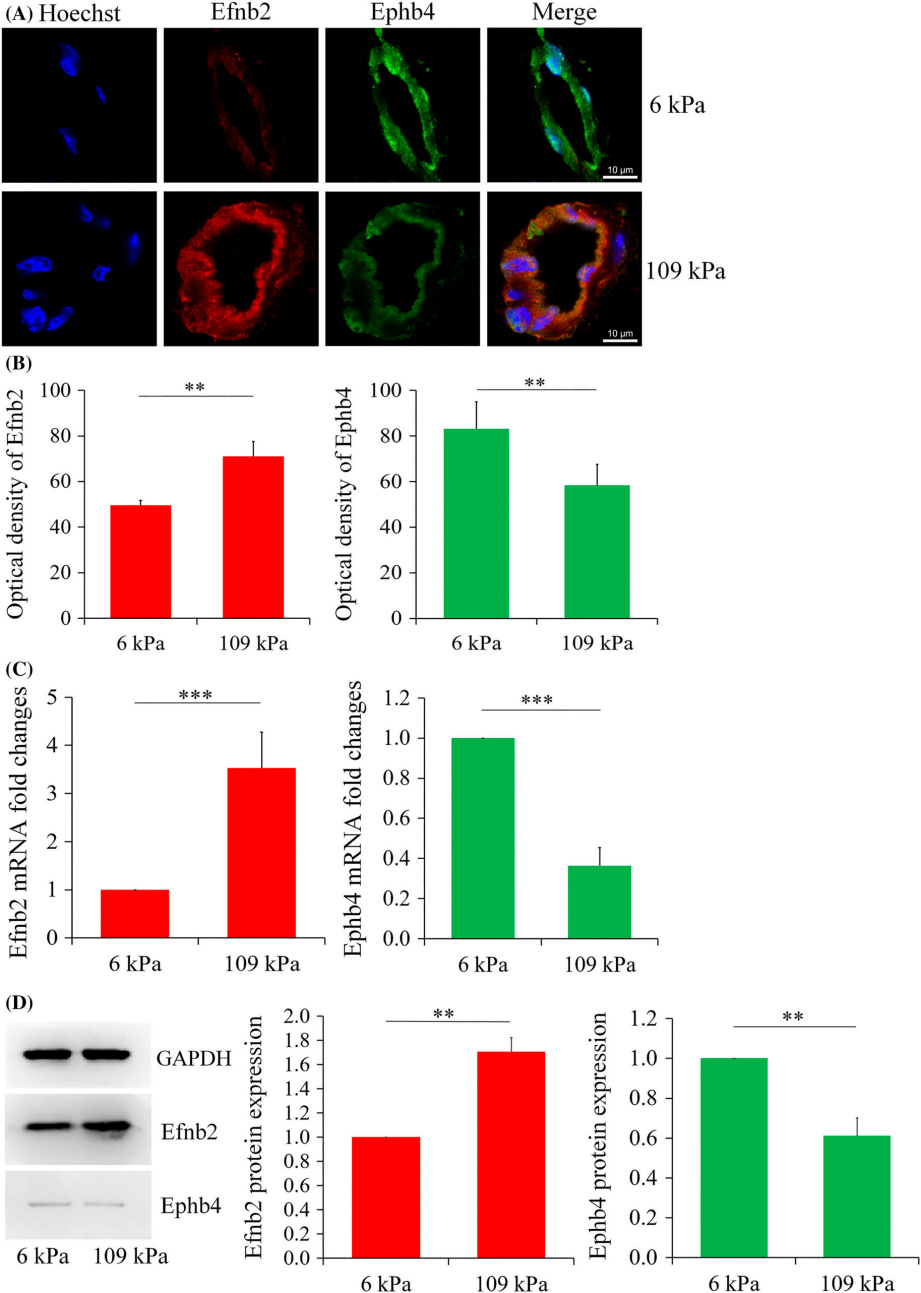
Monitoring arteriovenous endothelial markers in EPC‐loaded dextran‐based hydrogels in nude mice after 14 days. Two different dextran‐based hydrogels were fabricated with different stiffnesses (A; scale: 10 μm). Fluorescence intensity analysis (B). Data are expressed as mean ± standard deviation (SD). Real‐time polymerase chain reaction analysis of arteriovenous genes in newly generated vessels inside the two hydrogel types. Data were normalised to the housekeeping β‐actin gene and the control 6 kPa group (C). Monitoring protein levels of Efnb2 and Ephb4 in EPCs loaded in dextran hydrogels with different stiffnesses. *n* = 3. Student *t*‐test. *p < 0.05, **p < 0.01 and ***p < 0.001.^123^ Copyright 2019. Cell proliferation.

In an experiment, a stiffness‐adjustable dextran hydrogel was fabricated to monitor the angiogenesis behaviour of EPCs.^123^ To this end, maleimide‐modified dextran hydrogel with thiol‐reactive groups was mixed with mouse EPCs (1 × 10^6^/mL of culture medium), and angiogenesis factors VEGF, bFGF and IGF. The procedure was continued with the addition of a CD‐link (cross‐linker). Two types of dextran‐based hydrogels (2.0 and 7.0 mmol/L hydrogel) with different stiffness values, loaded cells and angiogenesis factors were transplanted into the nude mice. Data confirmed CD31^+^ cells using immunohistochemistry staining. It seems that these levels were more evident with the increase in dextran concentration and thioether bond content. Along with these findings, protein levels of Efnb2, an arterial EC biomarker, were more evident in highly concentrated dextran‐based hydrogel, whereas loaded EPCs tended to express Ephb4, a venous EC biomarker, in less concentrated dextran hydrogel after 14 days (Figure 6).^123^

**FIGURE 7:**
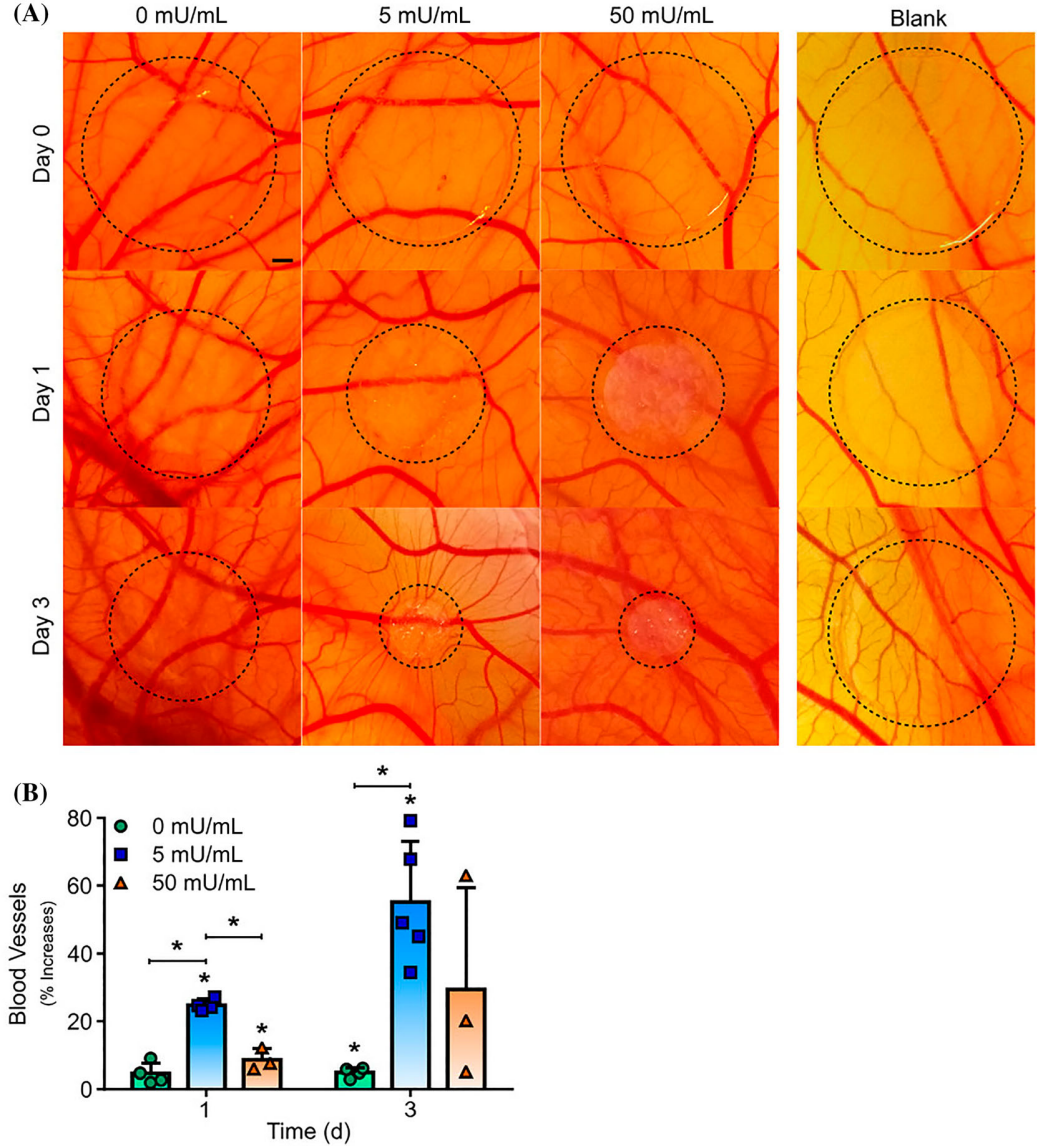
Angiogenesis properties of lyase (5 and 50 mU/mL)‐ and outgrowth EC‐loaded alginate hydrogel using an ex ovo chick chorioallantoic membrane assay (A, B). The formation of vascular units was stimulated in alginate hydrogel‐containing lyase 1 and 3 days after post‐transplantation (scale bar: 1 mm). Certain concentrations of alginate lyase (5 mU/mL) can efficiently stimulate angiogenesis (vascular density) in both time points compared to the control group (B; *n* = 3–5). One‐way analysis of variance with Tukey post hoc analysis. *p < 0.05.^119^ Copyright 2018. Biomaterials.

In an interesting study, Hirai et al. investigated intestinal end‐to‐end anastomotic healing using bFGF‐loaded Gel hydrogel sheets in rats.^177^ The hydrogel was cross‐linked using glutaraldehyde, and bFGF was loaded into the lyophilized hydrogel after being incubated with a growth factor solution. Data confirmed that the number of regenerated blood vessels was significantly stimulated by increasing the concentration of growth factor, in groups incubated with higher bFGF concentrations (i.e., 100 μg/mL). These features coincide with the increase of infiltrating fibroblasts (type I and III Col↑) at the site of injury.^177^ Some synthesis strategies have been used to increase the reciprocal interaction of incorporated EPCs with the supporting substrates. For example, Camci‐Unal et al. fabricated hyaluronic acid/heparin‐based methacrylated alginate hydrogels with a covalently immobilised CD34 antibody to stimulate cell adhesion and spreading using 1‐Ethyl‐3‐(3‐dimethylaminopropyl)carbodiimide (EDC)/N‐Hydroxysuccinimide (NHS) amine‐coupling method. Data confirmed that both EPCs and HUVECs attached to the modified surface and generated a cell layer compared to the non‐modified composites.^118^ It seems that the incorporation of different signalling molecules can stimulate the function of ECs within the hydrogels. In an experiment conducted by Su et al. basal membrane laminin‐derived YIGSR peptides or VEGF‐mimetic QK peptides were covalently added to PEG‐cross‐linked Gel hydrogel using PEG succinimidyl valerate. The culture of HUVECs or glomerular ECs on the surface of modified hydrogel‐induced cord‐like tubules stimulated the expression of critical cell surface proteins such as vWF, VE‐Cadherin (CD144), VEGFR‐2, CD31, integrin β1, Tie‐2, etc.^178^ The covalent attachment of angiogenesis factors into decellularized scaffolds seems another strategy to promote the dynamic function of EPCs.^179^ The covalent attachment of VEGF and Arg‐Gly‐Asp peptide to decellularized PEG‐cross‐linked porcine aortic valve leaflets promotes the proliferation of human cord blood EPCs (^3^H‐TdR^+^ cells↑) and the formation of single confluent EPC layer on the surface of the modified substrate. They found that the expression of tissue plasminogen activator and endothelial nitric oxide (eNOS) was also induced in primary cultured HUVECs on the modified surface.^179^ Of course, attention should be given to certain biodegradation properties of the hydrogel that are critical to regulating the angiogenesis potential of EPCs.^119^ Kevin and co‐workers fabricated charcoal‐activated alginate (2%) hydrogel and decorated it with RGD peptide using sulfo‐NHS/EDC to assess the dynamic angiogenesis rate. The final composites were enriched with different concentrations of alginate lyase (0, 5, 50 and 500 IU/mL). Data confirmed that the mesh size of final composites was increased over time by increasing alginate lyase concentration. The angiogenesis properties and migration of human outgrowth EPCs embedded inside an alginate lyase‐loaded hydrogel were induced in vitro and in vivo conditions compared to the control composite devoid of enzymatic compound (Figure 7).^119^

Both EPCs and stem cell byproducts can be simultaneously loaded within transplanted hydrogel for the induction of angiogenesis and different regenerative purposes. It was suggested that the injection‐embedded EPCs (7 × 10^5^/100 μL hydrogel) and EPC extracellular vesicles (EVs: 9.33 × 10^9^/100 μL hydrogel) using adamantane‐ and β‐cyclodextrin‐modified hyaluronic acid promoted the cardiac regeneration in a rat model of experimental myocardial infarction. Both EPC and/or EV‐loaded hyaluronic acid hydrogel can improve hemodynamic features and angiogenesis properties (vWF^+^, α‐SMA^+^ vascular units) in the infarcted rats compared to the matched control groups. Besides, the injection of EPC and/or EV‐loaded hydrogel led to the suppression of inflammatory response (CD11b↓), and fibrotic changes (Col fibres↓) after 4 weeks.^180^ In another strategy, the co‐transplantation of certain factors with vascular cells can improve the angiogenesis outcomes in the target tissues. For instance, Silva et al. used Arg‐Gly‐Asp‐conjugated macroporous oxidised alginate for the co‐delivery of human EPCs and/or outgrowth ECs with angiogenesis factor VEGF in SCID mice with ischemic hindlimb injury subjected to the ligation of femoral artery and vein.^181^ In hydrogel‐containing cells plus VEGF, necrotic changes and amputation possibility were reduced, coinciding with the significant increase in vascular density in the target sites compared to cell‐ and factor‐free control hydrogel and hydrogel supplemented with cells and/or VEGF.^181^

**FIGURE 8:**
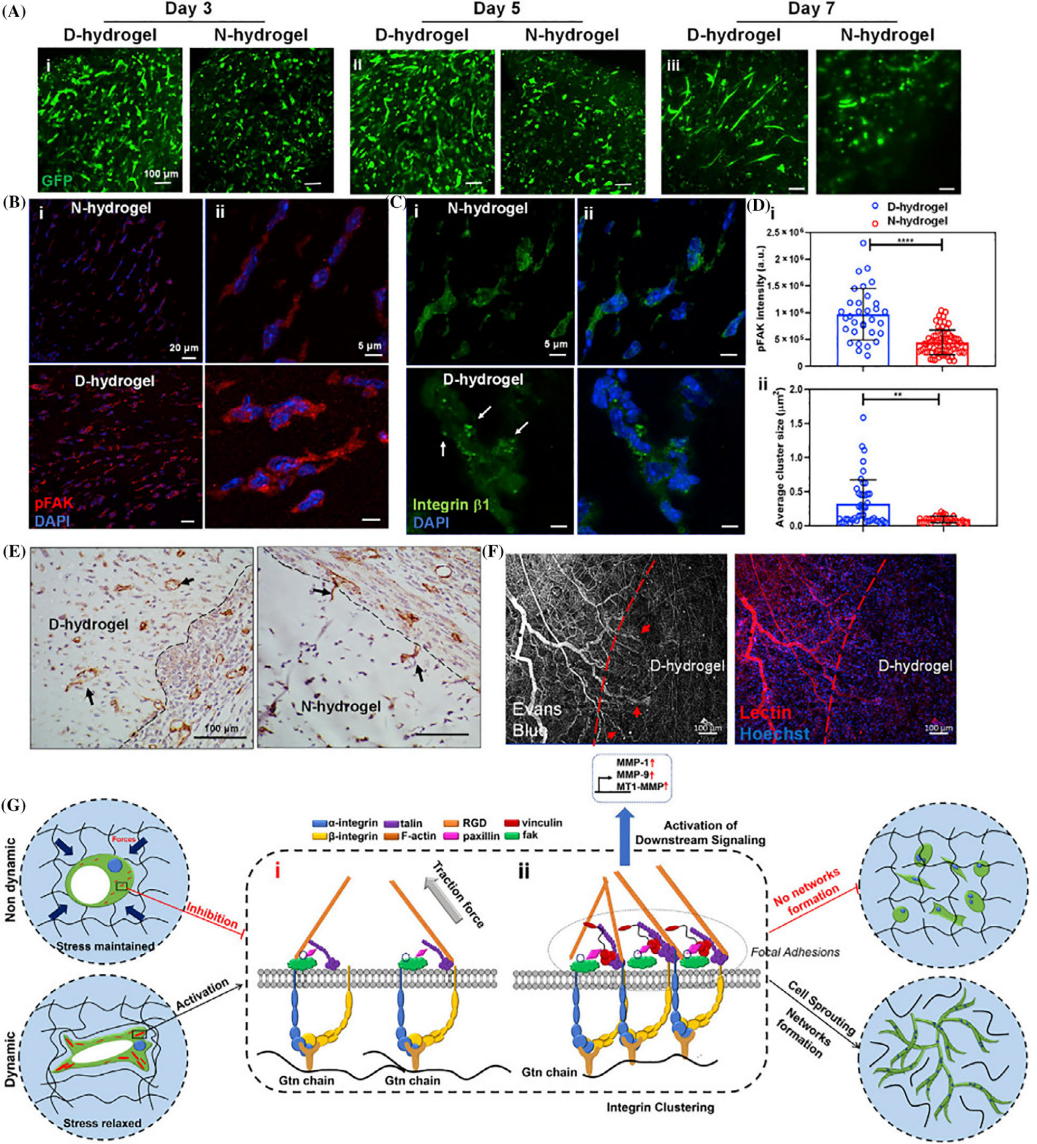
Human endothelial colony‐forming cell (ECFC) vasculogenesis behavior was monitored after being loaded in Gel/dextran hydrogels with dynamic (D‐hydrogel) and/or static (N‐hydrogel) covalent bonds in in vivo conditions (A‐G). Confocal images related to the subcutaneous transplantation of GFP+ ECFCs in nude mice and retrieval on days 3, 5, and 7 (A: *n* = 3). Data showed longer sprouts in the dynamic hydrogel group compared to the static hydrogel group. Monitoring phosphorylation of phosphorylated FAK (p‐FAK) in transplanted ECFCs on day 5 (B; Scale bars: 20 μm in section i and 5 μm in section ii). Along with tubulogenesis activity, data showed higher p‐FAK expression in ECFCs embedded into the dynamic hydrogel. Likewise, the cluster size of integrin increased in ECFCs embedded into dynamic hydrogel on day 5 (C). White arrows indicate green colored β1‐integrin (nuclei were stained with DAPI; Scale bars: 20 μm in section (i) and 5 μm in (ii). pFAK levels (i) and β1‐integrin cluster size (ii) (D). The injection of hydrogel in immunocompetence mice containing SDF‐1α indicated de novo vascular beds, and numerous infiltrating cells at the edge of D‐hydrogel on day 3 compared to N‐hydrogel. CD31+ infiltrated vascular units into hydrogel with dynamic and static covalent bonds (E; arrows; Scale bars, 100 μm). Determination of red‐colored lectin infiltrating blood vessels (red arrows) showed rapid in vivo vasculogenesis in the dynamic hydrogel. Evans blue dye was injected via the systemic route before the injecting hydrogels (F: Scale bars: 100 μm). **p < 0.01, and ***p < 0.0001. Engineered hydrogels with dynamic crosslinks lead to contractility‐related integrin clustering and FAK clustering without affecting hydrogel stiffness (G).^182^ Copyright 2020. Cell Stem Cell.

Along with hydrogel composition and the presence of certain growth factors, the dynamic contractibility of hydrogels can also affect the angiogenesis behaviour of EPCs.^182^ In an experiment, dynamic and static Gel/dextran hydrogel was applied for the evaluation of EPC growth and angiogenesis potential.^182^ The fabrication of dynamic Gel/dextran hydrogel was done based on the generation of covalent bonds between imine and acyl hydrazone, while the static hydrogel counterpart was synthesized using methacrylate.^182^ Data confirmed higher cytocompatibility, typical morphogenesis features and prominent tubulogenesis properties in embedded EPCs inside the Gel/dextran hydrogel with dynamic covalent bonds compared to the control composite with static covalent bonds. Protein levels of MMPs, FAK, phosphorylated FAK and clustering of integrin β1 were also stimulated in dynamic polymeric networks (Figure 8).^182^

## 4 CONCLUSION

EPCs are valid stem cell types for constructing vascular units and angiogenesis induction in in vitro and in vivo conditions. These cells can secrete angiocrine and/or directly mature into functional ECs. However, due to the distinct physicochemical properties of naïve hydrogels, further physical and chemical modifications can improve the angiogenesis potential in the targeted tissues. Co‐administration of EPCs with other cells and specific growth factors is also another strategy to increase vascularization in the transplants. It is suggested that future studies should focus on the evaluation of composites with different chemical formulas, laden cells and growth factors for the stimulation of angiogenesis in hypoxic/ischemic areas and other regenerative purposes.

## AUTHOR CONTRIBUTIONS

SR, ZA‐M, NDK, SAC, GR, HL and ES collected data and prepared the manuscript. GB and RR supervised the study and acquired the funding.

## CONFLICT OF INTEREST STATEMENT

The authors declare that they have no competing interests.
